# Beyond α-Tocopherol: Biology and Biotechnological Potential of Tocomonoenol and Tocodienol

**DOI:** 10.3390/biology15211917

**Published:** 2026-10-23

**Authors:** Qiao Ruan, Wajahat Hussain, Yuriy Balji, Yonghong Zhou

**Affiliations:** 1 Chongqing Chemical Industry Vocational College, Putidong Road 2009, Chongqing 401228, China; qiaoruanchemistry@outlook.com; 2 College of Agronomy and Biotechnology, Southwest University, Tiansheng Road 2, Chongqing 400715, China; wajahat7253@yahoo.com; 3 Department of Veterinary Sanitation, Faculty of Veterinary Sciences & Animal Husbandry, Saken Seifullin Kazakh Agro-Technical Research University, Astana 010011, Kazakhstan; yu.balji@kazatu.edu.kz; 4 LLP «NFT-KATU», Astana 010000, Kazakhstan

**Keywords:** vitamin E, tocomonoenol, tocodienol, tocochromanols, biosynthesis, antioxidant activity, metabolic engineering

## Abstract

Vitamin E-related tocochromanols include the rare homologs tocomonoenol (TM) and tocodienol (TD), containing one and two side-chain double bonds, respectively. This review critically evaluates their structural diversity, biosynthesis, natural occurrence, biological activities, and biotechnological potential. Recent analytical studies identify 11′-TM homologs, particularly 11′-α-TM and 11′-γ-TM, in plant oils and photosynthetic microorganisms, whereas 12′-α-TM, known as marine-derived tocopherol (MDT), is associated with cold-water marine organisms. Genetic evidence supports a VTE1/VTE2-dependent pathway for TM formation involving partially reduced prenyl precursors. Chemical partial hydrogenation of tocotrienols can generate TM and TD, while targeted microbial production remains a potential but unvalidated strategy. Cellular studies demonstrate the uptake and metabolism of 11′-TM, whereas antioxidant, anti-inflammatory, and adipocyte-related effects have been reported primarily for MDT. Preliminary evidence also suggests antiproliferative effects of α-TM in prostate cancer cell models, although therapeutic relevance remains unestablished. Importantly, the endogenous biosynthetic pathway of TD remains unresolved, and direct cellular and in vivo evidence for the biological activity of naturally occurring TD is lacking. Consequently, its physiological and therapeutic significance cannot yet be established. Future research should prioritize isotope tracing, candidate-enzyme validation, purified-compound pharmacokinetics, and compound-specific toxicological evaluation. Overall, TM shows emerging biological potential, whereas TD remains a major biosynthetic and functional knowledge gap.

## Simple Summary

Tocomonoenol and tocodienol are rare members of the vitamin E-related tocochromanol family and remain less studied than tocopherols and tocotrienols. Tocomonoenols occur in plant oils, microalgae, cyanobacteria, and marine organisms, whereas naturally occurring tocodienols are less extensively characterized. Their biological activities differ among individual forms. Cellular uptake and metabolism have been investigated for 11′-tocomonoenols, while antioxidant, anti-inflammatory, and adipocyte-related effects have primarily been reported for 12′-α-tocomonoenol, also known as marine-derived tocopherol. Direct biological evidence for naturally occurring tocodienols remains lacking. This review summarizes their structures, biosynthesis, natural sources, biological activities, and biotechnological potential, highlighting the major research gaps that must be addressed before their nutritional or therapeutic relevance can be established.

## 1. Introduction

Vitamin E research has evolved from the discovery of a dietary factor required for reproduction to the recognition of a chemically diverse group of plant-derived tocochromanols with important roles in membrane protection, cellular signaling, stress adaptation, and human nutrition. These compounds are synthesized predominantly by photosynthetic organisms, including higher plants, algae, and cyanobacteria, and enter animal and human tissues primarily through the diet [1,2]. In human nutrition, α-tocopherol remains the best-established form because it is selectively retained in the body and is capable of preventing the neurological manifestations of ataxia with vitamin E deficiency (AVED) [3]. At the same time, the terminology surrounding “vitamin E” remains under active discussion. Recent evaluations of vitamin E nomenclature have emphasized the need to distinguish the established vitamin activity of 2R-α-tocopherol from the broader group of structurally related tocochromanols [4].

All tocochromanols share a common structural architecture consisting of a chromanol head group bearing a phenolic hydroxyl group and a hydrophobic isoprenoid-derived side chain. Differences in chromanol-ring methylation generate the α-, β-, γ-, and δ-congeners, whereas the degree and position of side-chain unsaturation distinguish tocopherols, tocomonoenols (TM), tocodienols (TD), and tocotrienols [2,3]. To clarify the structural nomenclature of these compounds, Table 1 summarizes the chromanol-ring methylation patterns, side-chain double-bond positions, and accepted names of the major tocochromanol classes and their rare homologs. Historically, α-tocopherol has dominated nutritional and biomedical research, whereas γ- and δ-tocopherols and tocotrienols subsequently attracted attention because of their distinct antioxidant, anti-inflammatory, metabolic, neuroprotective, and anticancer properties [5,6,7,8]. The field has also progressed beyond viewing tocochromanols exclusively as chain-breaking antioxidants. Their metabolites can themselves exert biological effects; for example, long-chain vitamin E metabolites have been implicated in the regulation of inflammatory lipid mediators and signaling pathways, indicating that biological activity may depend on both the parent compound and its metabolic fate [9]. In parallel, advances in plant genetics, metabolic engineering, and synthetic biology continue to refine understanding of tocochromanol biosynthesis and are creating new opportunities for vitamin E biofortification and sustainable biomanufacturing [10].

Within this expanded framework, TM has emerged as a particularly intriguing but still underexplored class of tocochromanols. Naturally occurring 11′-TM homologs have been identified in pumpkin seed oil, palm-derived vitamin E fractions, cereals, kiwifruit, cyanobacteria, and several microalgal species, whereas 12′-α-TM, commonly termed marine-derived tocopherol (MDT), is characteristic of several cold-water marine organisms [11,12,13,14,15,16,17,18,19,20,21]. Importantly, recent analytical evidence suggests that TM may not be as exceptional in plant-derived foods as previously assumed. A 2025 gas chromatography–mass spectrometry (GC-MS) survey detected and quantified α- and/or γ-TM in 17 of 22 investigated edible plant-oil types, indicating a considerably broader distribution of these minor tocochromanols, although they generally remain present at relatively low concentrations [22]. This expanding distributional evidence further supports the need to reconsider TM as a biologically relevant minor component rather than merely an analytical curiosity.

Interest in TM has also increased because its biological profile is beginning to extend beyond occurrence and structural characterization. In HepG2 cells, 11′-α- and 11′-γ-TM undergo cellular uptake and metabolism to short-chain hydroxychroman derivatives, with differences observed between the two congeners [23]. MDT exhibits notable antioxidant performance under low-temperature conditions [14,24] and has demonstrated anti-inflammatory and adipocyte-related regulatory effects in cellular models [25]. More recently, α-TM was identified among serum metabolites differentiating prostate cancer from benign prostatic hyperplasia within the prostate-specific antigen gray zone. In separate experiments, α-TM inhibited proliferation and colony formation in androgen receptor-positive prostate cancer cells, with androgen receptor silencing abolishing the detectable growth-inhibitory response [26]. These findings extend the possible biological relevance of TM but remain preliminary and should not be interpreted as evidence of therapeutic efficacy in animals or humans.

TD represents an even larger knowledge gap. Naturally occurring 3′,11′- and 7′,11′-α-tocodienol positional isomers have been structurally characterized in palm oil and vitamin E-rich preparations using chromatographic enrichment, high-resolution mass spectrometry, and NMR spectroscopy [27,28]. However, compared with TM, information concerning TD remains extremely limited. Its natural abundance is very low, its endogenous biosynthetic pathway has not been experimentally resolved, and direct cellular or in vivo evidence for the biological activities of naturally occurring TD remains lacking [28,29].

Despite growing interest in rare tocochromanols, knowledge of TM and TD remains fragmented across analytical chemistry, plant biochemistry, food science, metabolism, and biomedical research. Recent vitamin E reviews have substantially advanced understanding of biosynthesis, nomenclature, metabolism, and emerging production technologies [9,10], but an integrated evaluation focused specifically on TM and TD remains needed. Accordingly, this review critically examines their structural diversity, biosynthetic origins, natural occurrence, biological activities, and biotechnological potential within the broader tocochromanol framework. Particular emphasis is placed on distinguishing experimentally established findings from chemically feasible, structurally inferred, or otherwise unvalidated mechanisms. By integrating current evidence and identifying major analytical, biosynthetic, functional, and translational gaps, this review aims to provide a framework for future investigation of these overlooked members of the tocochromanol family.

## 2. Structural Diversity and Biosynthetic Pathways

### 2.1. General Structure of the Vitamin E Family

All members of the vitamin E family share a fundamental structural architecture consisting of a polar chromanol ring and a hydrophobic isoprenoid side chain; together, these features govern their physicochemical behavior, membrane localization, and biological activities [2]. The chromanol ring bears a phenolic hydroxyl group that constitutes the reactive center for hydrogen-atom donation to free radicals and for interactions with other antioxidants in regenerative networks.

The methyl substitution pattern on the chromanol ring differentiates the four isomers within each subclass. The α-isomers carry three methyl groups at positions 5, 7, and 8; the β- and γ-isomers each carry two methyl groups (at positions 5,8 and 7,8, respectively); and the δ-isomers bear a single methyl group at position 8. This pattern directly influences the electron density of the phenolic ring and therefore modulates antioxidant reactivity: counter-intuitively, the δ-isomers with fewer methyl groups often display superior radical-scavenging capacity compared with α-isomers [30].

The 15-carbon isoprenoid side chain distinguishes the four subclasses:

Tocopherols carry a saturated phytyl chain. This structural stability makes them the predominant vitamin E form in virtually all photosynthetic tissues, with α-tocopherol reaching highest concentrations in photosynthetically active leaf chloroplasts.

Tocotrienols bear a geranylgeranyl chain containing three double bonds, a feature that influences membrane behavior and biological activity. Four isomers are recognized and are particularly enriched in monocot seeds (palm oil, rice bran oil) and in leaves of *Hypericum* species, where tocotrienols may constitute 13–32% of total vitamin E [31].

Tocomonoenol (TM) contains one double bond in its isoprenoid side chain. The 11′ and 12′ designations indicate the position of this double bond, whereas the α-, β-, γ-, and δ-prefixes denote differences in chromanol-ring methylation. Accordingly, 11′-TM encompasses different homologs, particularly 11′-α-TM and 11′-γ-TM, rather than representing a single compound. The 11′-α-TM homolog has been identified in palm-derived vitamin E fractions, microalgae, and cyanobacteria [17,20,21].

Additional β- and δ-TM homologs have been reported in plant matrices, although some trace-compound assignments remain tentative [15,32]. In contrast, marine-derived tocopherol (MDT) specifically refers to 12′-α-TM, which differs from 11′-α-TM in the position of its side-chain double bond and occurs in cold-water marine organisms [11,14]. In the Slovenian pumpkin cultivar *Slovenska golica*, γ-TM reaches 118.7 μg/g in roasted seed oil [12], whereas MDT accounts for 7.7–22.3% of total vitamin E in selected cold-water fish tissues [11]. The structures of isolated 11′-α-TM and 11′-γ-TM have been confirmed using NMR-based analyses [15,17].

Tocodienol (TD) carries two double bonds in the side chain and represents the most recently described member of the vitamin E family. Its structure was established by characteristic chemical shifts in ^1^H NMR spectra; the two recognized α-isomers bear double bonds at the 3′,11′ and 7′,11′ positions, respectively [28]. Both α-tocodienol isomers have been identified in palm oil and pumpkin seed oil, and traces are detectable in commercial vitamin E supplements [27,28].

A general structure–activity relationship in the vitamin E family relates higher side-chain unsaturation and fewer chromanol methyl groups to enhanced antioxidant capacity in certain experimental contexts, as demonstrated for δ-tocopherol and δ-tocotrienol relative to α-tocopherol [30]. In addition, tocopheryl fatty acid esters (TFAE) esterified derivatives of tocopherols found in peppers, walnuts, and related species appear to serve as a storage form of tocopherols in plants [33], though their direct relevance to TM and TD metabolism remains uncharacterized.

### 2.2. Biosynthesis of Tocopherols and Tocotrienols

The biosynthesis of vitamin E is confined to the chloroplasts of photosynthetic organisms and proceeds through two convergent precursor routes followed by a shared sequence of condensation, cyclization, and methylation reactions [34].

The aromatic ring precursor homogentisic acid (HGA) is derived from the tyrosine catabolism pathway. Tyrosine aminotransferase (TAT; encoded by *At5g53970* in *Arabidopsis thaliana*) converts tyrosine to 4-hydroxyphenylpyruvate, which is subsequently oxidized and decarboxylated by 4-hydroxyphenylpyruvate dioxygenase (HPPD) to yield HGA [35]. Loss-of-function of the *Arabidopsis TAT* gene results in significant accumulation of tyrosine and a marked reduction in tocopherol content. HPPD is also the enzymatic target of triketone herbicides; accordingly, overexpression of *HPPD* substantially elevates plant vitamin E levels while simultaneously conferring herbicide resistance [36]. Co-expression of yeast *Prephenate dehydrogenase* gene with *Arabidopsis HPPD* in tobacco leads to massive accumulation of tocotrienols in leaves [37]. In olive fruit, the expression of homogentisate phytyltransferase (HPT) and 2-methyl-6-phytylbenzoquinol (MPBQ) methyltransferase correlates closely with tocopherol content and is regulated by abiotic stress [38].

The isoprenoid side-chain precursor for tocopherols is phytyl diphosphate (PDP), derived primarily from the chlorophyll degradation product phytol. During chlorophyll breakdown, pheophytinase (PPH) and chlorophyll dephytylases (CLD1, CLD2, CLD3) release free phytol, which is subsequently phosphorylated sequentially by phytol kinase (VTE5) and phytyl phosphate kinase (VTE6) to generate PDP [39,40]. The farnesol kinase FOLK has been identified as a functional homolog of VTE5, and the *vte5-2 folk* double mutant completely abolishes tocopherol synthesis. In maize, phytol supply for tocopherol biosynthesis depends additionally on embryo-localized chlorophyll biosynthesis, with POR1 and POR2 serving critical roles in this pathway [41].

The unsaturated geranylgeranyl diphosphate (GGPP), which serves as the side-chain precursor for tocotrienols, is synthesized via the methylerythritol phosphate (MEP) pathway through the action of geranylgeranyl diphosphate synthase (GGPPS). In *Arabidopsis thaliana*, GGPPS11 is a major plastid isoform supplying GGPP for photosynthesis-related isoprenoids, including carotenoids, chlorophylls, tocopherols, phylloquinone, and plastoquinone [42]. However, wild-type *A. thaliana* lacks the dedicated homogentisate geranylgeranyltransferase (HGGT) required for the classical tocotrienol biosynthetic pathway and does not normally accumulate tocotrienols [40].

In the shared condensation step, HGA and PDP are coupled by homogentisate phytyltransferase (HPT; also designated VTE2) to yield 2-methyl-6-phytylbenzoquinol (MPBQ), the tocopherol precursor. Recent functional evidence in *Brassica napus* further demonstrated that modulation of *BnVTE2* expression produces corresponding changes in total tocopherol accumulation, supporting VTE2/HPT as an important control point in seed tocopherol biosynthesis [43]. Alternatively, HGA and GGPP are condensed by homogentisate geranylgeranyl transferase (HGGT) to give 2-methyl-6-geranylgeranylbenzoquinol (MGGBQ), the tocotrienol precursor. This condensation reaction thus constitutes the branch point between the tocopherol and tocotrienol pathways [44]. Deletion of the HPT homolog *slr1736* in *Synechocystis* abolishes tocopherol synthesis, confirming functional conservation across photosynthetic organisms [45]. Ectopic expression of the apple *MdHPT1* in tomato elevates tocopherol content in both leaves and fruits [46].

MPBQ and MGGBQ can follow two parallel routes that determine the final tocopherol and tocotrienol isomer composition. In one branch, MPBQ and MGGBQ are directly cyclized by tocopherol cyclase (VTE1) to form δ-tocopherol and δ-tocotrienol, respectively. Alternatively, MPBQ and MGGBQ are methylated by MPBQ/MGGBQ methyltransferase (VTE3) to generate 2,3-dimethyl-6-phytyl-1,4-benzoquinol (DMPBQ) and 2,3-dimethyl-6-geranylgeranyl-1,4-benzoquinol (DMGGBQ), respectively, which are subsequently cyclized by VTE1 to produce γ-tocopherol and γ-tocotrienol. VTE1 therefore catalyzes an essential cyclization step in tocochromanol biosynthesis; *vte1* mutants are deficient in tocopherols, whereas overexpression of *VTE1* in *Arabidopsis* increases total leaf tocopherol by more than sevenfold and shifts the tocopherol profile toward γ-tocopherol [47,48,49]. In the final methylation step, γ-tocopherol methyltransferase (γ-TMT; VTE4) converts δ-tocopherol and δ-tocotrienol to the corresponding β-isomers, whereas γ-tocopherol and γ-tocotrienol are converted to the corresponding α-isomer [50]. Two InDel markers within the maize *ZmVTE4* locus collectively explain 33% of the phenotypic variance in α-tocopherol content and have been deployed in marker-assisted selection [51]. Natural variation in soybean γ-TMT correlates with α-tocopherol content and provides additional targets for molecular breeding [52].

### 2.3. Spotlight: Structural Uniqueness and Putative Biosynthesis of Tocomonoenol and Tocodienol

Tocomonoenol and tocodienol occupy a chemically intermediate position within the vitamin E family. TM differs from tocotrienols by possessing only one, rather than three, side-chain double bonds; TD differs from both TM and tocotrienols in carrying exactly two double bonds. This gradation in unsaturation is not merely a chemical curiosity but has demonstrable functional consequences: it modulates membrane dynamics, influences radical-scavenging efficiency, and may affect the speed and efficiency of hepatic ω-oxidative catabolism.

The two positional isomers of TM differ in the location of the double bond along the side chain. In 11′-α-tocomonoenol the predominant plant and microalgal form the double bond resides at the 11′ position of the phytyl-like chain; in 12′-α-tocomonoenol (MDT), the double bond is shifted one carbon toward the methyl terminus. This subtle difference has been linked to differential antioxidant efficiency in cold-temperature environments: the 12′-isomer’s side-chain geometry enhances membrane fluidity in cold-water tissues, thereby improving the accessibility and regeneration kinetics of the chromanol-mediated radical-quenching reaction [24]. The two isomers are chemically distinguished by characteristic ^1^H NMR signals and GC/MS fragmentation patterns [13,15]. Although MDT belongs to the TM family, its biological activities should not be generalized to 11′-TM or other TM homologs without compound-specific experimental evidence.

For TD, structural elucidation has been achieved by countercurrent chromatography enrichment combined with high-resolution ^1^H NMR and high-resolution mass spectrometry. The 3′,11′-isomer and 7′,11′-isomer differ in the position of the more distal double bond along the side chain; both have been confirmed in palm oil and in commercial vitamin E preparations [27,28]. The coexistence of both TD isomers in the same oil matrices raises the possibility that they are generated by the same biosynthetic machinery from a common intermediate, though no enzymatic evidence is yet available.

Regarding the biosynthetic origin of TM, evidence supports at least one pathway involving condensation of homogentisic acid (HGA) with a partially reduced isoprenoid precursor. In *Arabidopsis thaliana*, biosynthesis of 11′- and 12′-γ-tocomonoenol was shown to depend on VTE1 and VTE2 and to be initiated by condensation of HGA with tetrahydrogeranylgeranyl pyrophosphate [53]. This provides direct genetic evidence for a TM biosynthetic route involving a partially reduced prenyl precursor. Another chemically feasible route involves the partial reduction of tocotrienol-type side chains. Chemical partial hydrogenation of α-tocotrienol generates substantial quantities of α-TM and α-TD, demonstrating the chemical feasibility of such conversion; however, this experiment does not establish that partial hydrogenation of preformed tocotrienols occurs as an endogenous biosynthetic mechanism in vivo [29]. In *Monodopsis subterranea*, 11′-α-TM accounts for approximately 5.5% of total tocochromanols under nitrogen-sufficient conditions. Nitrogen depletion markedly increases α-tocopherol accumulation without significantly affecting the absolute concentration of α-TM, suggesting distinct regulatory responses [19]. In addition, *Arabidopsis* WRI1 and DGAT1 mutants alter tocochromanol accumulation, indicating regulatory coupling between seed lipid metabolism and TM biosynthesis [53]. Chemical probe studies further demonstrated that activation of the GGPP reductase pathway can bypass chlorophyll-derived phytol dependency to directly enhance tocopherol synthesis, offering a conceptual template for understanding TM side-chain diversification [54].

The endogenous biosynthetic pathway of TD remains unresolved. Its side chain may arise from partially reduced geranylgeranyl-derived precursors or other modifications of isoprenoid intermediates, but these mechanisms remain hypothetical. No TD-specific biosynthetic enzyme has been identified, and its extremely low natural abundance complicates mechanistic investigations [28]. The novel chemical probe X57, which can simultaneously modulate tocopherol biosynthesis, competing pathways, and storage structures, represents a promising tool for dissecting TM and TD metabolic flux in future studies [54].

**Figure 1:**
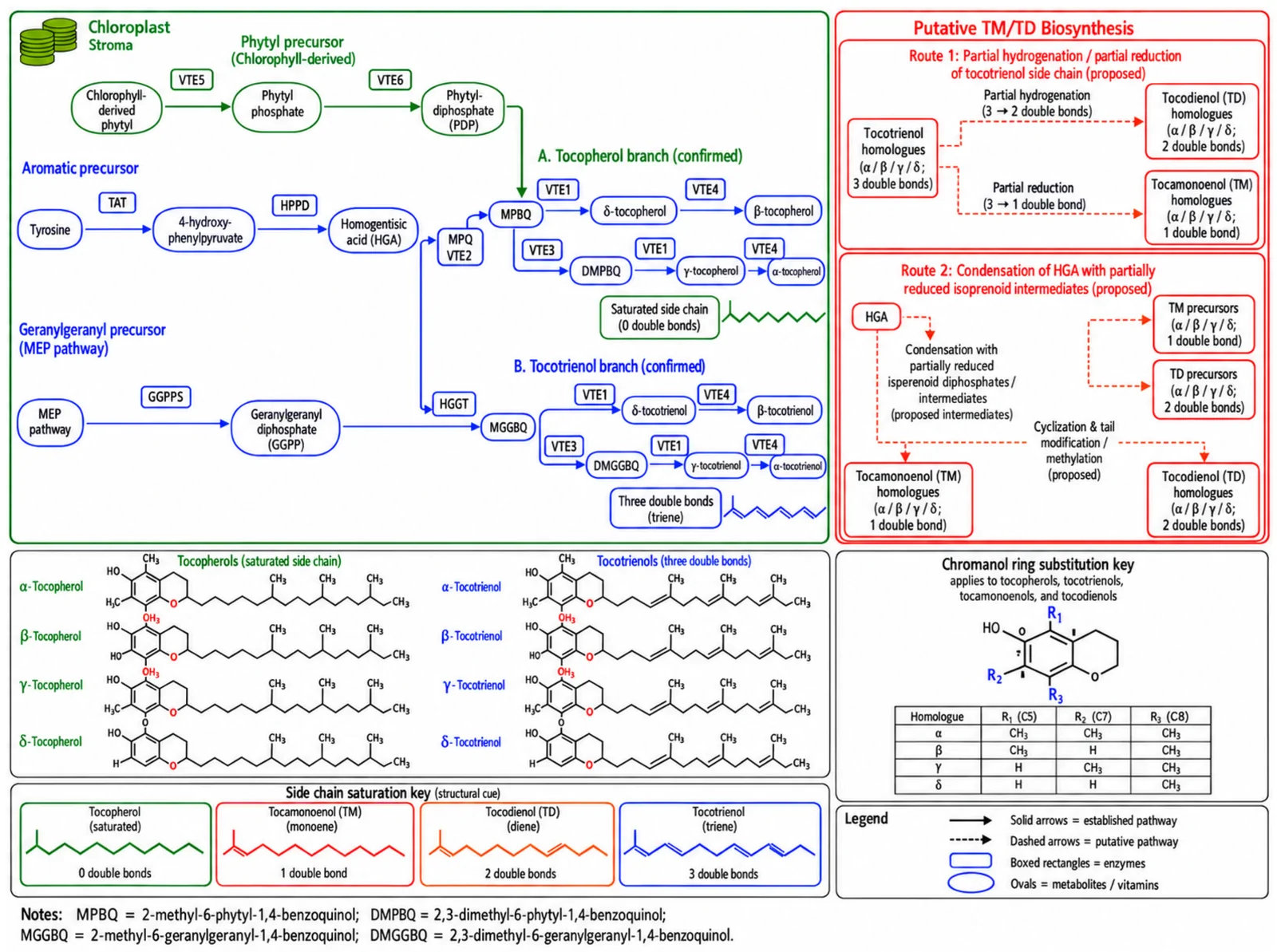
Established vitamin E biosynthetic pathways and proposed routes for tocomonoenol (TM) and tocodienol (TD) formation. Homogentisic acid (HGA) is condensed with phytyl diphosphate (PDP) by HPT/VTE2 or with geranylgeranyl diphosphate (GGPP) by HGGT. Subsequent cyclization and methylation involve VTE1, VTE3, and VTE4. A TM biosynthetic route involving partially reduced prenyl precursors has genetic support, whereas endogenous TD formation and proposed tocotrienol hydrogenation routes remain unconfirmed. Solid arrows indicate established reactions; dashed arrows indicate proposed pathways requiring experimental validation.

TM biosynthesis may be regulated differently in plants and microalgae, whereas TD regulation remains unresolved. In microalgae, nitrogen starvation experiments have demonstrated that TM levels remain stable while α-tocopherol increases sharply, implying independent regulation [19]. In pumpkin seed oil, roasting elevates γ-TM content, suggesting thermal stress may stimulate either de novo TM synthesis or conversion from tocotrienol precursors [12]. These observations collectively underscore that TM and TD are not merely structural curiosities or degradation artifacts but are metabolically regulated compounds whose physiological roles in the producing organisms remain to be fully elucidated. The established tocopherol and tocotrienol biosynthetic pathways, together with the proposed routes for TM and TD formation, are summarized schematically in Figure 1.

## 3. Natural Occurrence and Physicochemical Properties

### 3.1. Distribution in Plants, Algae, and Animals

Vitamin E is broadly distributed across all photosynthetic organisms, with species-specific and tissue-specific variation in both total content and isomer composition [55]. In most higher plant tissues, α-tocopherol is the dominant form in leaves, while γ-tocopherol predominates in many seeds, and tocotrienols are characteristic of monocot endosperms and certain dicot species.

In oilseed crops such as soybean, sunflower, and rapeseed, tocopherols constitute the bulk of total vitamin E. Black rice varieties contain markedly higher α-tocopherol, β-tocopherol, and α-tocotrienol than white or red rice, with total vitamin E ranging from 19.36 to 63.29 mg/kg [56], though neither TM nor TD has been detected in these cultivars. Pomegranate seed oil contains 362.69–397.48 mg total tocopherol per 100 g oil, with γ-tocopherol predominating and no trace vitamers detected [57]. In olive fruit, HPT and MPBQ methyltransferase gene expression closely tracks tocopherol content and responds to abiotic stress [38].

Tocotrienols show a more restricted distribution, being most abundant in palm oil, rice bran oil, and the leaves of certain *Hypericum* species, where they account for 13–32% of total vitamin E. Different drying conditions significantly affect tocotrienol retention in *Hypericum* leaves, a finding with implications for post-harvest processing of plants intended as vitamin E sources [31].

In microalgae and cyanobacteria, α-tocopherol is the predominant vitamer, though species-to-species variation is considerable. In *Tetraselmis* sp. and related flagellate microalgae, 11′-α-TM ranks as the second most abundant vitamer after α-tocopherol [20,21]. In *Monodopsis subterranea*, 11′-α-TM accounted for approximately 5.5% of total tocochromanols under nitrogen-sufficient conditions, whereas its relative proportion decreased following nitrogen depletion [19]. TD has not been detected in any cyanobacterial or microalgal species examined to date [20].

In terrestrial animals, vitamin E is not synthesized endogenously but is acquired from diet and stored in adipose tissue, liver, and muscle. Marine animals, especially cold-water species, contain or accumulate the 12′-α-tocomonoenol isomer known as marine-derived tocopherol. Cold-water fish and Antarctic krill accumulate MDT (12′-α-TM), which accounts for 7.7–22.3% of total vitamin E in selected tissues [11]. This accumulation may be associated with adaptation of membrane antioxidant systems to low temperatures [24]. MDT has also been detected in processed fish products at proportions similar to those in fresh tissue, with thermal stability comparable to α-tocopherol [13]. To date, TD has not been confirmed in any animal tissue.

### 3.2. Spotlight: Niche Sources of Tocomonoenol and Tocodienol

Pumpkin seed oil is arguably the richest and most well-characterized plant-based source of TM. In roasted oils from the Slovenian cultivar ‘Slovenska golica’, γ-tocomonoenol reaches 118.7 μg/g, with α-tocomonoenol also detectable [12]. The roasting-associated increase in γ-TM content is noteworthy and may reflect thermal conversion of tocotrienols; this possibility warrants direct experimental testing with isotopically labeled precursors.

Palm oil is one of the best-characterized plant sources of TD, and TD has also been reported in pumpkin seed oil. Both the 3′,11′- and 7′,11′-α-tocodienol isomers have been confirmed by countercurrent chromatography and high-resolution NMR [28], and α-TM is additionally present. Palm oil is simultaneously the principal commercial source of tocotrienols. Notably, marked inter-varietal variation exists: the total tocotrienol content of *Elaeis oleifera* Quepos reaches 890 μg/g oil [58], though the TD fraction consistently represents less than 1% of total vitamin E. This varietal diversity provides germplasm resources for genetic studies aimed at identifying determinants of TD accumulation.

Marine organisms represent the most significant biological reservoir of 12′-α-TM (MDT). Antarctic fish, Antarctic krill (*Euphausia superba*), and related cold-water taxa accumulate MDT as a quantitatively important vitamin E component adapted to low-temperature oxidative stress environments. In mice fed MDT-containing salmon roe oil, MDT accumulated preferentially in the liver and kidney compared with muscle and adipose tissue [59]. Fish liver shows the highest MDT concentrations, suggesting active accumulation and possibly a storage or signaling role at this site. When cold-water fish products are subjected to common food-processing treatments (frying, boiling, roasting), MDT degradation rates closely parallel those of α-tocopherol, indicating that existing food-processing parameters developed for α-tocopherol retention also adequately protect MDT [13].

Kiwifruit (*Actinidia deliciosa*) provides an additional plant source of δ-TM, with significantly higher concentrations in peel than in flesh [32], paralleling the general pattern of concentrated antioxidant deposition in outer protective tissues. TM has also been reported in sunflower oil, microalgae, and cereals, suggesting a broader natural distribution than previously recognized [16,60,61]. Systematic [61] surveys employing sensitive analytical platforms, including ultra-high-performance liquid chromatography coupled with high-resolution mass spectrometry (UHPLC–HRMS) and suitable chromatographic methods for isomer discrimination, across a wider range of oil crops, vegetables, and fermented plant products are warranted to map TM and TD occurrence comprehensively.

The currently documented natural sources, relative abundance, and analytical approaches used for the identification and characterization of TM and TD homologs are summarized in Table 2.

### 3.3. Analytical Challenges in the Identification and Quantification of TM and TD

The reliable identification and quantification of tocomonoenol (TM) and tocodienol (TD) remain analytically challenging because these compounds generally occur at much lower concentrations than conventional tocopherols and tocotrienols. Their low abundance, particularly in complex plant oils and biological matrices, can complicate chromatographic detection, isolation, and structural characterization. Enrichment procedures, including countercurrent chromatography and preparative separation, have therefore been employed to obtain sufficient quantities for detailed structural analysis [15,17,28].

An additional challenge is the discrimination of structural and positional isomers. For example, 11′-α-TM and 12′-α-TM (marine-derived tocopherol, MDT) differ only in the position of their side-chain double bond, while 3′,11′-α-TD and 7′,11′-α-TD contain the same number of double bonds at different positions. Such compounds may exhibit similar chromatographic behavior and mass-spectral characteristics, making unequivocal identification difficult using conventional high-performance liquid chromatography (HPLC) or gas chromatography–mass spectrometry (GC–MS) alone. High-resolution mass spectrometry can support molecular-formula assignments, but it does not necessarily establish double-bond positions. Accordingly, isolation followed by nuclear magnetic resonance (NMR) spectroscopy provides stronger evidence for positional-isomer confirmation, as demonstrated in structural studies of rare TM and TD homologs [15,17,28].

The limited availability of authentic TM and TD reference standards further complicates analytical validation and quantitative comparisons among studies. Quantification based on structurally related tocopherol or tocotrienol standards may introduce uncertainty because detector responses and recovery characteristics cannot automatically be assumed to be identical. In addition, complex sample matrices may cause chromatographic interference or matrix-dependent analytical responses. Extraction, saponification, concentration, and derivatization procedures may also introduce analyte losses or chemical alterations unless appropriately controlled. These concerns are especially relevant when interpreting trace peaks, as previous investigations of complex vitamin E preparations have highlighted the possibility of analytical artifacts and tentative assignments [60].

Future studies should therefore employ validated extraction and recovery procedures, matrix-matched calibration where appropriate, and authentic standards whenever available. Complementary techniques, including HPLC with fluorescence detection, high-resolution LC–MS, GC–MS, and NMR spectroscopy, should be combined according to the analytical objective. Importantly, tentative detection, MS-supported identification, authentic-standard-assisted quantification, and NMR-confirmed structural characterization should be reported as distinct levels of analytical evidence. Such methodological transparency is essential for accurately defining the natural distribution, abundance, and structural diversity of TM and TD.

## 4. Biological Functions and Health Benefits

### 4.1. Background: Antioxidant and Anti-Inflammatory Functions of Tocopherols and Tocotrienols

Tocopherols and tocotrienols are established lipid-soluble antioxidants that protect biological membranes from oxidative damage. Their chromanol hydroxyl group can donate a hydrogen atom to lipid peroxyl radicals, interrupting lipid peroxidation chain reactions [1]. Beyond antioxidant activity, certain tocopherols and tocotrienols have been investigated for their effects on inflammatory signaling, including pathways associated with nuclear factor κB (NF-κB), cytokine production, and lipid mediator metabolism [6,62]. In plants, tocopherols contribute to protection against photooxidative stress and lipid peroxidation, as demonstrated in *Arabidopsis thaliana* mutants deficient in tocopherol biosynthesis [63,64].

These established functions provide a biochemical framework for investigating TM and TD because both compounds share the characteristic chromanol head group. However, structural similarity does not establish equivalent biological activity. Differences in side-chain unsaturation and double-bond position may influence membrane interactions, metabolism, and biological responses. Accordingly, antioxidant and anti-inflammatory findings reported for conventional tocopherols and tocotrienols should be regarded as background evidence rather than direct demonstrations of TM or TD activity [2,3].

### 4.2. Background: Metabolic and Disease-Related Evidence for Established Tocochromanols

Research on tocopherols and tocotrienols has identified a range of biological effects beyond their classical antioxidant functions. In experimental systems, particular tocotrienol homologs have been associated with regulation of cholesterol metabolism, inflammatory signaling, cellular proliferation, and responses to oxidative stress [5,6]. Investigations of tocotrienols have also explored neuroprotective and bone-related outcomes, although the strength of evidence varies according to the compound, experimental model, and measured endpoint [7,65,66]. In plants, tocopherols contribute to stress tolerance and seed longevity through protection against oxidative deterioration of cellular components [63,64].

These findings are relevant to TM and TD primarily as biological context and as a basis for formulating experimentally testable hypotheses. They cannot establish that TM or TD produces comparable metabolic, anticancer, neuroprotective, or other health effects. In particular, clinical and animal findings obtained with tocopherols, tocotrienols, or tocotrienol-rich preparations should not be extrapolated to purified TM and TD without direct investigation. The available evidence for these rare homologs is considered separately in the following subsection, with attention to specific chemical forms, experimental models, and limitations.

### 4.3. Compound-Specific Biological Evidence for TM, MDT, and TD

Unlike conventional tocopherols and tocotrienols, TM and TD have not been evaluated across a comparable range of experimental systems. Available findings must therefore be interpreted separately for individual 11′-TM homologs, 12′-α-TM (MDT), and mixed tocochromanol preparations. In HepG2 cells, 11′-α-TM and 11′-γ-TM undergo cellular uptake and metabolism to hydroxychroman derivatives, providing evidence of cellular processing but not establishing clinical antioxidant or therapeutic effects [23]. By contrast, reports of antioxidant activity under low-temperature conditions primarily concern MDT [14,24]. Moreover, biological responses observed with partially hydrogenated tocotrienol mixtures cannot be assigned to individual TM or TD homologs without compound-specific testing [67]. Direct biological evidence for naturally occurring TD remains particularly limited, and its proposed functions are largely based on structural analogy rather than experimental validation [28,29].

To distinguish the strength and relevance of available findings, Table 3 presents a compound-specific evidence framework for 11′-TM homologs, 12′-α-TM (MDT), and TD. Although MDT belongs to the TM family, it should be considered separately in biological evaluations because its double-bond position and experimental evidence differ from those of 11′-TM. Importantly, structural confirmation of a compound does not establish biological efficacy, and observations from mixed preparations cannot be attributed to individual homologs without further investigation.

MDT (12′-α-TM) stands apart from all other vitamin E vitamers in its exceptional antioxidant performance at low temperatures. Its superior cold-environment efficiency is mechanistically linked to the geometry of the 12′-double bond, which increases the conformational freedom of the side chain within low-temperature membrane phospholipid bilayers, enhancing the accessibility of the chromanol head group to membrane-generated lipid peroxyl radicals and facilitating more rapid regeneration from the tocopheroxyl radical form by water-soluble co-antioxidants [24]. This structural adaptation constitutes a compelling example of structure-guided bioactivity and is consistent with the ecological distribution of MDT being restricted to cold-water marine organisms.

The anti-inflammatory bioactivity of MDT has been directly demonstrated in macrophage and adipocyte models. MDT significantly reduces LPS-induced secretion of IL-6 and TNF-α in RAW264.7 macrophages at concentrations lower than those required for equivalent suppression by α-tocopherol or γ-tocopherol [25]. In 3T3-L1 adipocytes, MDT treatment decreases secretion of IL-6 and monocyte chemoattractant protein-1 (MCP-1) while upregulating the anti-inflammatory adipokine adiponectin, pointing to potential utility in obesity-associated adipose tissue inflammation [25].

Evidence for metabolic regulation differs among individual TM homologs and experimental preparations. In 3T3-L1 adipocytes, 12′-α-TM (MDT) increased PPARγ expression, promoted adipocyte differentiation, and enhanced adiponectin production [25]. In diabetic–obese KK-Ay mice, a partially hydrogenated tocotrienol-rich preparation containing multiple tocochromanol homologs improved fasting blood glucose and cholesterol profiles [67]. However, these effects cannot be attributed to an individual TM or TD homolog. In HepG2 cells, 11′-α-TM exhibited distinct metabolic behavior compared with tocopherol and tocotrienol [23], although its systemic bioavailability and metabolic benefits remain unestablished.

The neuroprotective properties of MDT appear related to its superior bioavailability in neural tissue relative to β-tocopherol [68], although direct mechanistic investigation of TM neuroprotection is lacking. The existing evidence from tocotrienol models provides a conceptual basis for expecting that TM, with its intermediate unsaturation level, may share the blood–brain barrier permeability advantages of tocotrienols over tocopherols.

A recent serum metabolomic study identified α-tocomonoenol (α-TM) among the metabolites differentiating patients with prostate cancer from those with benign prostatic hyperplasia within the prostate-specific antigen (PSA) gray zone (4–10 ng/mL), with higher serum levels observed in the prostate cancer group [26]. However, this association does not establish a causal relationship or demonstrate a protective or disease-promoting role for α-TM. In separate cellular experiments, α-TM inhibited proliferation and colony formation in LNCaP and 22Rv1 prostate cancer cells, while no significant reduction in RWPE-1 prostate epithelial cell viability was observed under the tested conditions [26]. Androgen receptor (AR) silencing abolished the detectable growth-inhibitory response, suggesting the involvement of AR-associated signaling. Nevertheless, these findings are limited to specific cell-line models and cannot establish efficacy under physiological conditions. Furthermore, although molecular docking predicted a possible interaction between α-TM and AR, direct molecular binding has not been experimentally confirmed. The precise positional-isomer identity of α-TM should also be established before extrapolating these findings to specific 11′-TM or 12′-TM homologs.

As regards TD, direct experimental data on its biological activity are absent. Two lines of indirect inference suggest meaningful bioactivity: (1) a linear positive correlation between side-chain double bond number and antioxidant capacity has been observed within vitamin E homolog series, predicting that TD would exceed TM but fall short of tocotrienol in radical-quenching efficiency; and (2) a synthetic δ-tocodienol analog, prepared by semisynthetic modification, displayed superior radical-scavenging activity relative to δ-tocotrienol in cell-free assays [69]. However, neither in vitro cellular models nor in vivo studies of TD bioactivity are currently available, and this constitutes the most significant knowledge gap in the vitamin E field today. The structural analogy to tocotrienols combined with the observed biological diversity across the vitamin E family justifies systematic functional characterization of TD as a high research priority.

**Figure 2:**
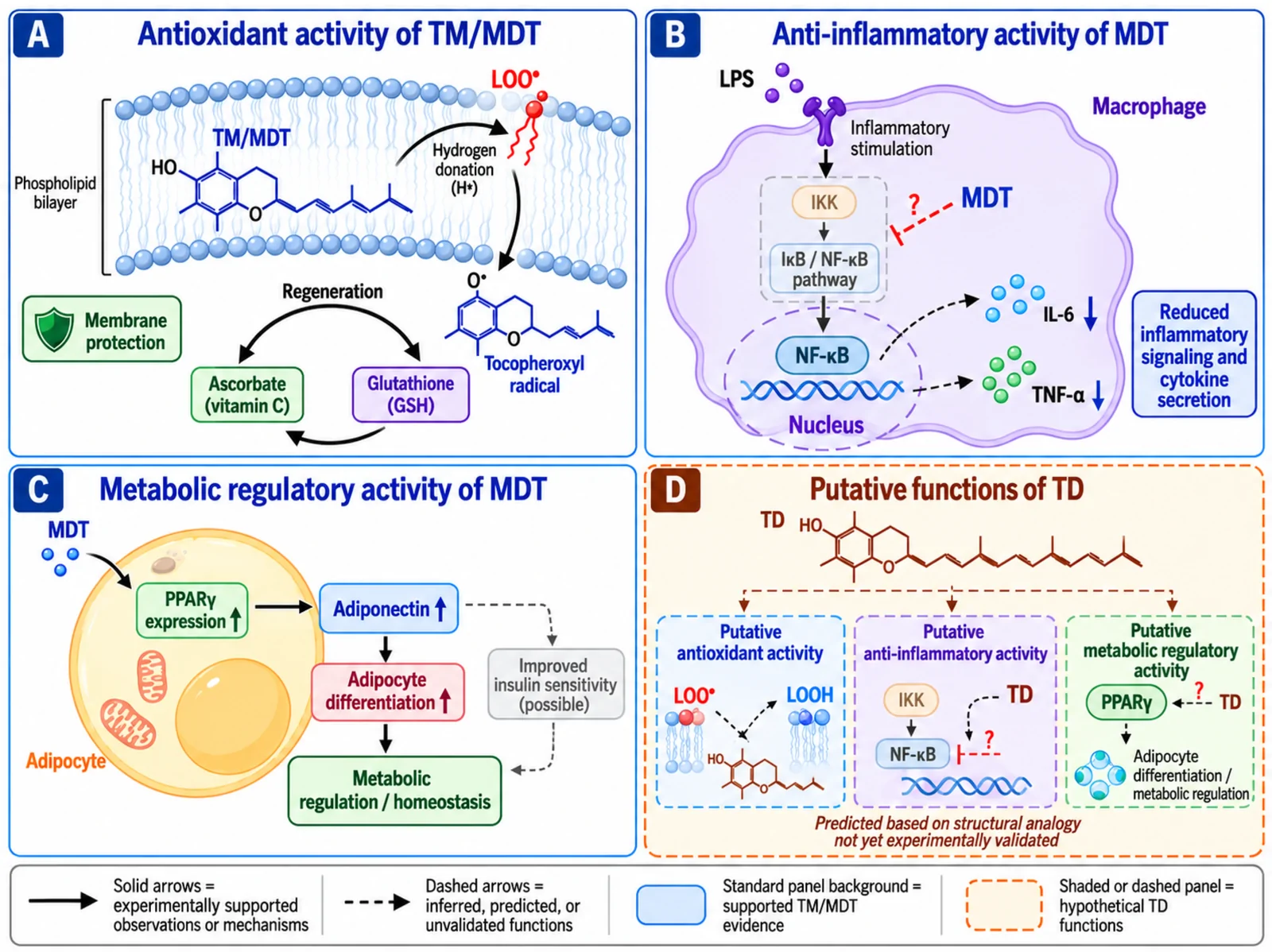
**Experimental and proposed biological activities of tocomonoenol (TM), marine-derived tocopherol (MDT), and tocodienol (TD).** (**A**) Proposed membrane antioxidant mechanism of TM, with low-temperature antioxidant evidence primarily for MDT. (**B**) MDT-associated suppression of inflammatory cytokines in macrophages; NF-κB involvement remains hypothetical. (**C**) MDT-mediated regulation of PPARγ, adiponectin, and adipocyte differentiation; improved insulin sensitivity remains unconfirmed. (**D**) Putative antioxidant, anti-inflammatory, and metabolic activities of TD inferred from structural analogy. Solid arrows indicate experimentally supported effects; dashed arrows indicate proposed mechanisms. LOO•, lipid peroxyl radical; LPS, lipopolysaccharide; GSH, glutathione; IKK, IκB kinase; NF-κB, nuclear factor κB; PPARγ, peroxisome proliferator-activated receptor γ.

The potential for pharmacological synergy between TM and other bioactive compounds has not been directly investigated and therefore remains an open area for future research. The tocotrienol-rich fraction from oat supplementation significantly improved glycemic, blood pressure, and lipid parameters in metabolic syndrome patients [70], providing clinical evidence for the metabolic effects of tocotrienol-rich preparations, although these findings cannot be directly extrapolated to TM or TD. A tocotrienol-rich fraction was also found to attenuate lipid dysregulation in nonalcoholic fatty liver disease by modulating purine metabolism [71]. The available evidence for TM homologs, including MDT-specific anti-inflammatory and adipocyte-related effects, together with the proposed but unvalidated biological activities of TD, is summarized in Figure 2.

## 5. Biotechnological Strategies for Production and Fortification

### 5.1. Metabolic Engineering in Crops

Comprehensive understanding of the vitamin E biosynthetic pathway has enabled targeted manipulation of both total content and isomer composition in numerous crop species [72,73,74,75]. While current strategies focus primarily on tocopherols and tocotrienols, they establish a technical and conceptual framework applicable to future TM and TD engineering.

Overexpression of *HPT* represents one of the most reproducible strategies for elevating total tocopherol content. Constitutive expression of *AtHPT1* in *Arabidopsis* increased total leaf vitamin E by 4.4-fold and seed content by 40% [76]. Overexpression of barley *HGGT* in maize increased total seed vitamin E by up to 6-fold, with a substantial shift toward tocotrienols [77], demonstrating that redirection of side-chain precursor supply can dramatically alter vitamer profiles. Ectopic expression of apple *MdHPT1* in tomato elevates tocopherol in both leaves and fruits [46], and *HPPD* overexpression doubles seed vitamin E content in tobacco [36]. The coordinated co-expression of yeast prephenate dehydrogenase with Arabidopsis HPPD drives tocotrienol accumulation in tobacco leaves [37].

Optimization of isomer composition for enhanced nutritional value has been achieved principally through manipulation of γ-TMT (VTE4). Overexpression of γ-TMT in soybean, perilla, and rapeseed efficiently diverts the γ-tocopherol flux toward α-tocopherol; in soybean seeds, α-tocopherol content was elevated by 11.1–18.9-fold [78,79]. Plastid-targeted overexpression of Atγ-TMT in *Arabidopsis* concurrently enhances tolerance to salt and heavy-metal stress [80]. Overexpression of *BnaC02.TMT.a* in rapeseed simultaneously elevates α-tocopherol, modifies fatty acid composition, and improves salt stress tolerance [81].

Molecular marker-assisted selection has provided cost-effective strategies for breeding vitamin E content. Two InDel markers in *ZmVTE4* explain 33% of phenotypic variance in maize α-tocopherol content [51]. In rapeseed, candidate gene association analysis identified polymorphisms in *BnaX.VTE3.a* and *BnaA.PDS1.c* as significantly associated with tocopherol content and composition, and derived CAPS markers have been deployed for rapid screening of high vitamin E germplasm [82,83]. Genome-wide association analysis in wheat identified 27 quantitative trait loci (QTLs) associated with tocopherol content, with Qδ.toc.6A fine-mapped to a 5.26 Mb interval, providing a basis for candidate gene identification [84].

For enhancement of MEP pathway flux, overexpression of 1-deoxy-D-xylulose-5-phosphate synthase (*DXS*), the rate-limiting enzyme in isoprenoid precursor supply, has been shown to cooperatively increase vitamin E content when combined with downstream gene overexpression [85]. Potential engineering approaches include reducing competition for GGPP from carotenoid biosynthesis and modifying phytol availability through chlorophyll turnover. However, their effectiveness for targeted TM or TD production remains unverified [39].

For TM and TD, targeted metabolic engineering remains a proposed strategy rather than an experimentally validated production approach. Potential directions include screening pumpkin and palm germplasm for variation in TM and TD accumulation, comparing transcriptomic and metabolomic profiles of microalgal strains with contrasting TM contents, and identifying candidate enzymes involved in the formation of partially reduced isoprenoid side-chain precursors. The involvement of such enzymes, including putative reductases, must be established through biochemical assays, genetic analysis, and isotope-tracing experiments before they can be considered validated engineering targets. Studies in microalgae indicate that nitrogen availability can substantially alter α-tocopherol accumulation without producing a corresponding increase in TM [19], suggesting that cultivation-based enhancement of TM cannot yet be assumed. Although engineered Saccharomyces cerevisiae has been used to produce δ-tocotrienol at a reported titer of 3262.2 μg/L [86], this result demonstrates production of δ-tocotrienol rather than TM or TD. Developing analogous systems for TM and TD would require additional pathway characterization, enzyme validation, and experimental confirmation of the target products.

### 5.2. Microbial Synthesis and Future Prospects

Microbial engineering has demonstrated the feasibility of producing certain established tocochromanols, particularly tocotrienols. For example, Han, Wu [86] reconstructed a δ-tocotrienol biosynthetic pathway in *Saccharomyces cerevisiae* using modular pathway engineering and fermentation optimization, achieving a reported titer of 3262.2 μg/L. However, this finding should not be interpreted as direct evidence for TM or TD production or for the scalability of their biosynthesis. The principal technological gap is the lack of sufficiently characterized and validated pathways for generating the partially unsaturated side-chain precursors required for targeted TM and TD formation, particularly the positional isomers of TD.

One potential future approach would involve identifying enzymes capable of generating prenyl precursors with defined degrees and positions of unsaturation. Isoprenoid-chain reductases related to enzymes involved in geranylgeranyl diphosphate metabolism represent possible candidates, but their suitability for TM or TD production remains hypothetical [39]. Comparative genomic and biochemical investigations of TM-accumulating organisms, including *Tetraselmis* species and other microalgae, may help identify relevant candidate genes. These candidates would require experimental validation through enzyme assays, targeted genetic manipulation, and heterologous expression before their roles in TM or TD biosynthesis could be established.

CRISPR-Cas9-mediated modification of tocochromanol biosynthetic genes also represents a potential future engineering strategy. Genes encoding homogentisate phytyltransferase (HPT/VTE2), tocopherol cyclase (VTE1), and γ-tocopherol methyltransferase (VTE4), together with as-yet-unidentified side-chain-modifying enzymes, could be investigated as candidate targets. Although manipulation of established vitamin E biosynthetic genes can modify tocopherol or tocotrienol accumulation [73,81], their capacity to selectively enhance TM or TD production remains unverified. Accordingly, proposed genome-editing and microbial-engineering strategies should be considered research hypotheses until supported by compound-specific analytical confirmation, metabolic-flux studies, and reproducible production data.

Advanced delivery systems developed for lipophilic vitamin E compounds, including nanoemulsions, vesicular carriers, transfersomal systems, and solid lipid nanoparticles, provide a potential framework for future TM and TD formulations [87,88,89]. Given the very low natural abundance of TM and TD, such approaches may help maximize the utility of the limited quantities available for biological and clinical investigation. These formulation technologies can enhance the aqueous dispersibility, oral bioavailability, and potentially the targeted delivery of minor vitamin E vitamers to specific tissues, enabling pharmacologically meaningful dosing from limited quantities of purified material.

## 6. Conclusions and Future Perspectives

The tocochromanol family comprises structurally related compounds that differ in chromanol-ring methylation and side-chain unsaturation. Tocopherols and tocotrienols have been intensively investigated, whereas TM and TD remain comparatively underexplored. Their intermediate degrees of side-chain unsaturation provide an important opportunity to understand how structural variation influences biosynthesis, membrane behavior, metabolism, and biological activity.

TM is now recognized across a broad range of biological sources, including cyanobacteria, microalgae, higher plants, edible oils, and cold-water marine organisms. The 11′-TM forms occur predominantly in photosynthetic organisms, whereas 12′-α-TM, or marine-derived tocopherol (MDT), is particularly associated with cold-adapted marine species. Experimental studies demonstrate cellular uptake and metabolism of TM, while MDT exhibits notable antioxidant activity and experimentally supported anti-inflammatory and adipocyte-related effects. Recent in vitro evidence also suggests an androgen receptor-associated antiproliferative effect of α-TM, although its physiological and therapeutic significance remains unresolved. Importantly, metabolic effects reported in diabetic–obese mice were obtained using a hydrogenated tocotrienol-rich preparation containing multiple minor tocochromanols and therefore cannot be attributed specifically to TM. By contrast, direct cellular and in vivo evidence for naturally occurring TD remains largely absent.

Progress has also been made in understanding TM biosynthesis. Genetic evidence supports at least one VTE1/VTE2-dependent pathway involving condensation of homogentisic acid with a partially reduced prenyl precursor. Chemical partial hydrogenation can also generate TM and TD, demonstrating chemical feasibility but not proving that this route operates naturally. The enzymes and precursor pathways responsible for endogenous TD formation remain unresolved.

The following research priorities are essential for advancing the understanding of TM and TD, with particular emphasis on resolving TD biosynthesis and establishing compound-specific biological functions.

TD biosynthesis and candidate-enzyme validation: Determining the endogenous biosynthetic pathway of TD should be a primary research priority. Stable-isotope tracing of potential isoprenoid precursors, combined with comparative transcriptomics, metabolomics, reverse genetics, and biochemical assays, may help identify the reactions responsible for generating its partially unsaturated side chain. Candidate enzymes should be tested for substrate specificity, catalytic activity, and their ability to produce structurally defined TD intermediates. Such studies are necessary because the enzymes and metabolic pathways responsible for natural TD formation remain unresolved [28].

Compound-specific biological functions: The biological activities of purified 11′-TM homologs, 12′-α-TM (MDT), and individual TD positional isomers should be investigated separately using standardized cellular and animal models. Particular attention should be given to antioxidant responses, inflammatory regulation, metabolic signaling, and potential adverse effects. Although cellular studies have provided evidence for TM uptake and metabolism and for MDT-associated biological responses, these findings cannot be generalized to all TM homologs [24,25]. For naturally occurring TD, direct functional characterization remains a major unmet research need.

Heterologous pathway reconstruction and production: Once candidate biosynthetic enzymes have been identified, their functions should be evaluated through heterologous expression and pathway reconstruction in suitable microbial or plant systems. Metabolic-flux analysis and compound-specific analytical verification will be necessary to establish whether these systems produce the intended TM or TD positional isomers. Although engineered yeast has been used to produce δ-tocotrienol [86], successful biosynthesis and scalable production of TM and TD remain to be demonstrated.

Purified-compound pharmacokinetics and bioavailability: Future investigations should determine the absorption, distribution, metabolism, and elimination of individual TM and TD homologs using purified compounds and, where feasible, stable-isotope-labeled materials. Particular emphasis should be placed on tissue accumulation, metabolic transformation, and differences between 11′-TM and MDT. Cellular metabolic findings should be validated in appropriate animal models before conclusions regarding systemic bioavailability or physiological relevance are drawn [24].

Compound-specific toxicology and translational evaluation: Systematic toxicological assessment of purified TM and TD homologs is required before potential nutritional or therapeutic applications can be considered. Priority should be given to dose–response relationships, cellular toxicity, metabolic safety, and repeated-exposure studies in appropriate experimental models. Such evaluations are particularly important for TD because direct biological and safety data remain insufficient. Human intervention studies should follow, rather than precede, rigorous structural characterization, pharmacokinetic evaluation, and preclinical safety assessment.

Analytical validation and natural distribution: These mechanistic and biological investigations should be supported by sensitive analytical methods, authentic reference standards, and structural confirmation of rare positional isomers. Expanded surveys of plants, microalgae, marine organisms, and other biological sources will help identify suitable materials for studying TM and TD formation and obtaining sufficient quantities of purified compounds for experimental evaluation.

TM and TD expand the structural diversity of the tocochromanol family, but their levels of biological characterization remain substantially different. TM has progressed from structural identification toward experimentally supported biosynthetic and biological characterization, whereas TD remains primarily an analytical and biosynthetic frontier. Advances in high-resolution analytics, isotope tracing, multi-omics, reverse genetics, and synthetic biology should now make it possible to resolve these gaps and determine whether these rare tocochromanols have meaningful nutritional, physiological, or biotechnological applications.

## Figures and Tables

**Table 1:** Structural classification and nomenclature of major tocochromanol groups, including rare tocomonoenol and tocodienol homologs.

Tocochromanol Group	Number of Side-Chain Double Bonds	Double-Bond Position(s)	α-, β-, γ-, and δ-Forms	Accepted Names/Synonyms
Tocopherols	0	None	α, β, γ, δ	Tocopherols; saturated phytyl side chain
11′-Tocomonoenols	1	11′	α and γ structurally characterized; β and δ reported with more limited structural evidence	11′-TM
12′-Tocomonoenol	1	12′	α confirmed; other forms not established	12′-α-TM; marine-derived tocopherol (MDT)
Tocodienols	2	3′,11′ or 7′,11′	α positional isomers structurally confirmed; other forms require further verification	TD; 3′,11′-α-TD; 7′,11′-α-TD
Tocotrienols	3	3′,7′,11′	α, β, γ, δ	Tocotrienols; geranylgeranyl side chain

Notes: The α-, β-, γ-, and δ-designations indicate methyl substitution patterns on the chromanol ring: α (C5, C7, C8), β (C5, C8), γ (C7, C8), and δ (C8). Prime symbols (′) identify positions along the isoprenoid side chain. TM, tocomonoenol; TD, tocodienol; MDT, marine-derived tocopherol. The structural identification of some rare TM and TD homologs remains tentative and requires further confirmation.

**Table 2:** Natural occurrence, distribution, and analytical characterization of tocomonoenol and tocodienol homologs.

Rare Vitamer/Isomer	Source and Matrix	Reported Occurrence/Abundance	Analytical Method and Identification Confidence	Reference
11′-α-TM and 11′-γ-TM	Pumpkin (*Cucurbita pepo* L. ‘*Slovenska golica*’); roasted seed oil	α-TM: 17.6 ± 0.6 μg/g oil; γ-TM: 118.7 ± 1.0 μg/g oil. In the same oil, γ-tocotrienol was only 6.9 ± 0.2 μg/g.	GC-MS-supported identification; HPLC quantification using reference materials.	[12]
11′-γ-TM, 11′-β-TM, δ-TM; α- and γ-TD positional isomers	Unrefined organic pumpkin seed oil prepared from seeds roasted at approximately 110 °C	γ-TM was isolated as 6.8 mg at 96.0% purity from approximately 2 L of oil. The study also detected 3′,11′- and 7′,11′-α-TD and corresponding γ-TD isomers, together with β-TM and δ-TM.	11′-γ-TM isolated and structurally confirmed by NMR; additional trace TM/TD homologs tentatively assigned using GC–MS and retention behavior.	[15]
11′-α-TM	Palm-oil vitamin E-rich extract/palm-derived dietary supplement	Approximately 30 mg α-TM was enriched at ~75% purity from 1 g crude material; subsequent purification yielded 5.6 mg at 99.5% purity. A trace of 12′-α-TM was also observed.	11′-α-TM isolated by chromatography and structurally confirmed by ^1^H NMR; GC–MS used for purity assessment.	[17]
α-TM	Mesocarp oils from six Costa Rican palm genotypes (*Elaeis guineensis*, *E. oleifera*, and an O × G hybrid)	α-TM was detected as a minor constituent. The *Elaeis oleifera* Quepos genotype contained 890 μg/g oil of total tocotrienols and 892 μg/g oil of total vitamin E. These values represent total tocochromanol measurements, not α-TM concentrations.	HPLC-FLD quantification and GC–MS-supported identification; positional-isomer confirmation by NMR not reported.	[58]
3′,11′-α-TD and 7′,11′-α-TD	Palm-oil-derived tocotrienol-rich fraction and crude palm oil	An α-TD trace component was first identified in a palm-derived tocotrienol-rich fraction; subsequent enrichment demonstrated two approximately equally represented α-TD positional isomers in palm oil.	Initial α-TD assignment by high-resolution MS; subsequent NMR confirmation of 3′,11′- and 7′,11′-α-TD after chromatographic enrichment.	[27,28]
δ-TM	Kiwifruit (Actinidia chinensis); peel and pulp	δ-TM was present predominantly in the fruit peel and at lower abundance in the pulp.	δ-TM isolated and structurally characterized using mass spectrometry and 1D/2D NMR.	[32]
11′-α-TM	Aquatic photosynthetic organisms, including *Tetraselmis* sp. and *Nannochloropsis oceanica*	*Tetraselmis* sp.: 15.20 ± 3.03 mg/kg DW, corresponding to ~17% of total tocochromanols. *N. oceanica*: 2.46 ± 0.71 mg/kg DW (~0.6%). In these organisms, α-TM was the second most abundant tocochromanol after α-tocopherol.	HPLC–FLD quantification with LC-MS^n^- and GC-MS-supported 11′-α-TM identification.	[21]
11′-α-TM	Cultured *Chlorella sorokiniana*, *Nannochloropsis limnetica*, and *Tetraselmis suecica*	HPLC-FLD values were 0.82 ± 1.43 mg/kg DW in *C. sorokiniana*, below HPLC-FLD quantification in *N. limnetica*, and 5.5 ± 4.83 mg/kg DW in *T. suecica*. α-TM represented <1% of total tocochromanols in these cultures.	HPLC-FLD quantification; 11′-α-TM assignment supported by LC–MS^n^ and GC–MS.	[20]
11′-α-TM	*Monodopsis subterranea* biomass	α-TM was 7.64 ± 1.90 mg/kg DW (~5.5% of total tocochromanols) under nitrogen sufficiency and 6.11 ± 0.97 mg/kg DW (~1.5%, calculated from group means) after nitrogen depletion. Depletion significantly increased α-tocopherol but not α-TM.	HPLC-FLD quantification; LC–MS- and GC-MS-supported 11′-α-TM identification.	[19]
MDT (12′-α-TM)	Antarctic notothenioid fish tissues, Antarctic krill (*Euphausia superba*), and phytoplankton	MDT represented 2.8–22.3% of total vitamin E across Antarctic samples. The highest MDT proportion reported was 22.3% in liver of *Champsocephalus gunnari*; liver of *Gobionotothen gibberifrons* contained 416.7 pmol/mg wet tissue total vitamin E with 7.7% MDT.	HPLC-based identification and quantification of MDT; no independent NMR confirmation reported in this study.	[11]
11′-α-TM and 11′-γ-TM	Commercial edible plant oils: 29 samples representing 22 plant species	α-TM and/or γ-TM were detected and quantified in 17 of 22 oil types. Examples included α-TM in almond oil (3.8 mg/kg) and γ-TM in argan oil (18 mg/kg) and chia oil (3.2 mg/kg). TMs were detected more frequently than tocotrienols in this survey.	GC–MS identification and quantification of α-/γ-TM using purified reference standards and selected-ion monitoring.	[22]

**Table 3:** Comparative evidence framework for 11′-tocomonoenol (TM) homologs, 12′-α-tocomonoenol (MDT), and tocodienol (TD).

Evidence Category	11′-TM Homologs	12′-α-TM (MDT)	TD
Structural identity and purified material	11′-α-TM and 11′-γ-TM have been isolated and structurally characterized using chromatographic and NMR-based methods [15,17]	A distinct α-TM positional isomer associated with cold-water marine organisms; MDT has been investigated separately in biological studies [11,25]	The naturally occurring 3′,11′-α-TD and 7′,11′-α-TD positional isomers have been structurally characterized, but their biological activities remain largely untested [28].
Cellular evidence	Uptake and metabolism of 11′-α-TM and 11′-γ-TM have been examined in HepG2 cells [23].	Anti-inflammatory responses have been reported in RAW264.7 macrophages, and adipocyte-related effects have been observed in 3T3-L1 cells [25].	No direct cellular bioactivity evidence for naturally occurring TD has been established.
Animal evidence	Compound-specific functional evidence for purified 11′-TM remains insufficient.	Dietary availability and tissue distribution have been investigated in mice, including studies using MDT-containing dietary materials [59,68]	No biological effects have been independently established for purified TD. Results from partially hydrogenated tocotrienol mixtures cannot be attributed specifically to TD [67]
Human evidence	No controlled human intervention evidence establishing biological efficacy for individual 11′-TM homologs.	No controlled human intervention evidence establishing biological efficacy for MDT.	No human intervention evidence.
Evidence inferred from structural analogy	Potential antioxidant and membrane-related functions may be proposed from similarities to other tocochromanols, but require homolog-specific validation.	Proposed low-temperature membrane mechanisms should be distinguished from directly measured biological outcomes [24].	Antioxidant and other biological functions remain hypothetical. Cell-free findings involving a synthetic TD analog do not establish the activity of naturally occurring TD [69].

## Data Availability

No new data were created or analyzed in this study. Data sharing is not applicable to this article.

## Abbreviations

CCC, countercurrent chromatography; DW, dry weight; GC-MS, gas chromatography–mass spectrometry; HPLC, high-performance liquid chromatography; HPLC–FLD, high-performance liquid chromatography with fluorescence detection; LC–MS, liquid chromatography–mass spectrometry; NMR, nuclear magnetic resonance; TM, tocomonoenol; TD, tocodienol; MDT, marine-derived tocopherol.
